# Comparison of ^68^Ga-PSMA-11 PET-CT with mpMRI for preoperative lymph node staging in patients with intermediate to high-risk prostate cancer

**DOI:** 10.1186/s12967-017-1333-2

**Published:** 2017-11-07

**Authors:** Qing Zhang, Shiming Zang, Chengwei Zhang, Yao Fu, Xiaoyu Lv, Qinglei Zhang, Yongming Deng, Chuan Zhang, Rui Luo, Xiaozhi Zhao, Wei Wang, Feng Wang, Hongqian Guo

**Affiliations:** 1 Department of Urology, Drum Tower Hospital, Medical School of Nanjing University, Institute of Urology, Nanjing University, 321 Zhongshan Rd., Nanjing, 210008 Jiangsu People’s Republic of China; 2 Department of Nuclear Medicine, Nanjing First Hospital, Nanjing Medical University, Nanjing, 210006 China; 3 Department of Pathology, Drum Tower Hospital, Medical School of Nanjing University, Nanjing, 210008 Jiangsu People’s Republic of China; 4 Department of Radiology, Drum Tower Hospital, Medical School of Nanjing University, Nanjing, 210008 Jiangsu People’s Republic of China

**Keywords:** Prostate cancer, PSMA, PET-CT, mpMRI, Lymph node staging

## Abstract

**Background:**

To evaluate the diagnostic value of ^68^Ga-PSMA-11 PET-CT with multiparametric magnetic resonance imaging (mpMRI) for lymph node (LN) staging in patients with intermediate- to high-risk prostate cancer (PCa) undergoing radical prostatectomy (RP) with pelvic lymph node dissection (PLND).

**Methods:**

We retrospectively identified 42 consecutive patients with intermediate- to high-risk PCa according to D′Amico and without concomitant cancer. Preoperative ^68^Ga-PSMA-11 PET-CT, pelvic mpMRI and subsequent robot assisted laparoscopic RP with PLND were performed in all patients.

**Results:**

Among 42 patients assessed, the preoperative PSA value, Gleason score, pT stage and intraprostatic PCa volume of patients with LN metastases were all significantly higher than those without metastases (*P* = 0.029, 0.028, 0.004, respectively). The average maximum standardized uptake value (SUV) of ^68^Ga-PSMA-11 PET-CT positive PCa of patients with or without LN metastases were 13.10 (range 6.12–51.75) and 7.22 (range 5.4–11.2), respectively (P < 0.001). ^68^Ga-PSMA-11 PET-CT and pelvic mpMRI had the ability of succeed on preoperative definite accurate diagnosis and accurate localization of primary PCa in all 42 patients. Fifteen patients (35.71%) had a pN1 stage. 51 positive LN were found. Both ^68^Ga-PSMA-11 PET-CT and pelvic mpMRI displayed brillient patient-based and region-based sensitivity, specificity, negative predictive value and positive predictive value. There was no statistical difference for the detection of LNMs according to the diameter of the LNMs between ^68^Ga-PSMA-11 PET-CT and mpMRI in this study.

**Conclusions:**

Both ^68^Ga-PSMA-11 PET-CT and mpMRI performed great value for LN staging in patients with intermediate- to high-risk PCa undergoing RP with PLND. However, despite excellent performance of ^68^Ga-PSMA-11 PET-CT, it cannot replace mpMRI that remains excellent for lymph node staging.

## Background

Prostate cancer (PCa) is the most common tumor in men around the United States and Europe [1]. Radical prostatectomy (RP) is the most widely used form of treatment for patients with localized PCa [2]. Lymph node (LN) status is an important prognostic factor for a newly diagnosed PCa [3]. Current guidelines recommend computed tomograph (CT) or conventional magnetic resonance imaging (MRI, T1W and T2W sequences) to evaluate metastatic spread to LN prior to RP, especially for intermediate- or high-risk PCa [4]. However, these staging modalities depend solely on morphologic information and LN involvement is mainly assessed by size [5]. Thus, these techniques yielded relatively poor specificity for evaluating LN status.

Multiparametric MRI (mpMRI) with its best soft tissue resolution plays an important role in the management of patients with PCa [6]. It contains T1- and T2-high-resolution weighted imaging, diffusion-weighted imaging (DWI), dynamic contrast-enhanced (DCE) and other functional MRI techniques. DWI provided information on tissue cellular density and membrane integrity while DCE enabled visualization of vascular permeability and perfusion [7]. The incorporation of these newer sequences allows functional tissue information to supplement the anatomic information provided by T1W and T2W sequences [7], which was widely used for PCa diagnosis, localization, staging. It is believed that mpMRI is more accurate for PCa diagnosis and LN status assessment than conventional MRI (T1W and T2W sequences). Thus, it was considered to be a promising technique for LN status assessment.

Recently, ligands of the prostate-specific membrane antigen (PSMA) have been introduced in positron emission tomography (PET) imaging of PCa, targeting an extracellular domain of this transmembraneous cell-surface protein [8, 9]. PSMA is almost exclusively expressed in prostate tissue and often shows a substantial overexpression on PCa cells [8, 10]. PET-CT using the radiolabelled PSMA inhibitor [^68^Ga] Glu-urea-Lys(Ahx)- HBED-CC (^68^Ga-PSMA-11) has the potential to improve the sensitivity and specificity for preoperative LN staging. However, the comparative value of pelvic mpMRI and ^68^Ga-PSMA-11 PET-CT for the LN staging of PCa is still unknown. Therefore, the purpose of our retrospective analysis was to evaluate the diagnostic value of ^68^Ga-PSMA-11 PET-CT in comparison to pelvic mpMRI for LN staging in patients with intermediate- to high-risk PCa undergoing RP with pelvic lymph node dissection (PLND).

## Methods

### Patients

From March 2017 to July 2017, forty-two consecutive patients with intermediate- to high-risk PCa according to D′Amico and without concomitant cancer who underwent ^68^Ga-PSMA-11 PET-CT imaging, pelvic mpMRI and subsequent robot assisted laparoscopic RP with PLND at our institution were included into the study. Seventeen (40.48%) patients had intermediate risk and 25 (59.52%) patients had high risk PCa at PLND. All patients gave written informed consent for the purpose of anonymized evaluation and publication of their data. The retrospective analysis was approved by the Ethics Committee of the Drum Tower Hospital, Medical School of Nanjing University (Reference: DT2017028).

### MpMRI examination and analysis

Pelvic mpMRI was performed with a 3.0-T MR scanner (Achieva 3.0 T TX, Philips Medical Systems, The Netherlands) by using a 16-channel phased array coil as described previously [11, 12]. Transverse/coronal/sagittal (18 slices, thickness 3 mm/gap 0.5 mm, TR 3744 ms, TE 120 ms, number of signals acquired 2, resolution 1.49 mm × 1.51 mm) T2-weighted turbo spin-echo (TSE) images were acquired. DWI, spin-echo echo-planar images (18 slices, thickness 3 mm, intersection gap 1 mm, TR 925/TE 41 ms, number of signals acquired 1, resolution 3 mm × 3 mm, b-factor 0/800/1500 s/mm^2^) were acquired. And T1 high-resolution isotropic volume with fat suppression after gadolinium injection was employed for DCE images (133 slices, thickness 3 mm, no intersection gap, TR 3.1/TE 1.46 ms, number of signals acquired 1, resolution 1.49 mm × 1.51 mm, dynamic scan time 00:06.9). Mappings of the ADC were generated from b 0, b 800 and b 1500 images of DWI using the Philips WorkStation software (Extended Workspace, EWS). All MRI scans were reviewed by an experienced radiologist with no prior clinical information. LNs were classified as malignant if lying in the territory of drainage of prostatic tumors and either if they were oblong with a short-axis diameter > 10 mm or were rounded with a short-axis > 8 mm or if they showed restricted diffusion on the DWI and ADC map or increased contrast enhancement.

## ^68^Ga-PSMA-11 PET-CT examination and analysis

The ^68^Ga-PSMA-11 was synthesized with an ITG semi-automated module (Germany, Munich) as described previously [13]. ^68^Ga-PSMA-11 was stable in vitro, its radiochemical purity was > 99% after 2 h of radiolabeling. All patients underwent PET-CT in a uMI 780 PET-CT scanner (United Imaging Healthcare (UIH), Shanghai, China) 60 min after intravenous injection of ^68^Ga-PSMA-11 (median, 131.72 MBq, range 130.6–177.6 MBq). First, a CT scan (130 keV, 80 mAs) was obtained without using contrast medium. Static emission scans, corrected for dead time, scatter and decay, were acquired from the vertex to the proximal legs. This required the patient assume 4 bed positions with 2 min per bed position. The images were iteratively reconstructed and included CT-based attenuation correction with the OSEM algorithm using 4 iterations with 8 subsets and Gaussian filtering to an in-plane spatial resolution of 5 mm at full-width at half-maximum. For calculation of the standardized uptake value (SUV), circular regions of interest were drawn around the area with focally increased uptake in transaxial slices and automatically adapted to a three-dimensional volume of interest using UIH WorkStation software (United Imaging Healthcare) at 50% isocontour. Datasets were fully corrected for random coincidences, scatter radiation, and attenuation. Images were interpreted by three experienced nuclear medicine physicians based on visual assessment. Final decisions were reached by consensus. A positive scan was defined as one showing abnormal focal increases in tracer activity within a lesion with an intensity level higher against a surrounding background considered to be malignant. The uptake of ^68^Ga-PSMA-11 was quantified SUV ^max^.

### Surgery

All patients underwent robot assisted laparoscopic RP with a bilateral meticulous template PLND (dissection of all nodes surrounding the common iliac, external iliac, and internal iliac vessels, in the obturator fossa, and in the presacral region). The LN specimens were prospectively mapped and labelled separately on a standardized LN map according to the anatomic regions just listed and sent for histopathologic examination. Para-aortic and pararectal nodes were removed only if positive sentinel LNs were found.

### Histologic examination

The pathologist received the labeled LNs on the standardized LN map, as well as the prostate gland. The prostate gland was sectioned at 3 mm intervals by using a whole-mount technique. Tissue slices were then formalin fixed, paraffin embedded, and microtome cut. Similarly, LNs were fixed overnight in 4% formalin to dissolve the fatty tissue. The LN stations were examined by palpation, visual inspection, and sectioning. The lamellated LNs were processed and paraffin embedded. These paraffin blocks were serially sectioned until the whole LN was cut. Both prostate gland and LN sections were stained with hematoxylin–eosin (HE) and examined by light microscopy by an experienced uropathologist. Disease positivity was defined as the presence of any metastatic deposits in a LN. Pathologic TNM stage was determined according to the 2010 TNM classification. The prostatic tumor volume was calculated as the sum of the tumor areas on HE slides (cm^2^) × section thickness (0.3 cm) × 1.33.

### Statistical analysis

All demographic data, including continuous and variables, was analyzed by independent Chi square test. Positive LNs were categorised in nine anatomical fields according to their origin (confer surgery). Positive LNs at imaging were recorded on the same standardised LN map. The histopathologic analysis of the LNs served as the reference to which the corresponding anatomic sites on preoperative imaging were compared. Sensitivity, specificity, positive predictive value (PPV), and negative predictive value (NPV) for LN status of ^68^Ga-PSMA-11 PET-CT and mpMRI were calculated, respectively. *P* < 0.05 were considered significant. Statistical analysis was carried out with SPSS v. 21 (SPSS INC., Chicago, Ill).

## Results

**Fig. 1:**
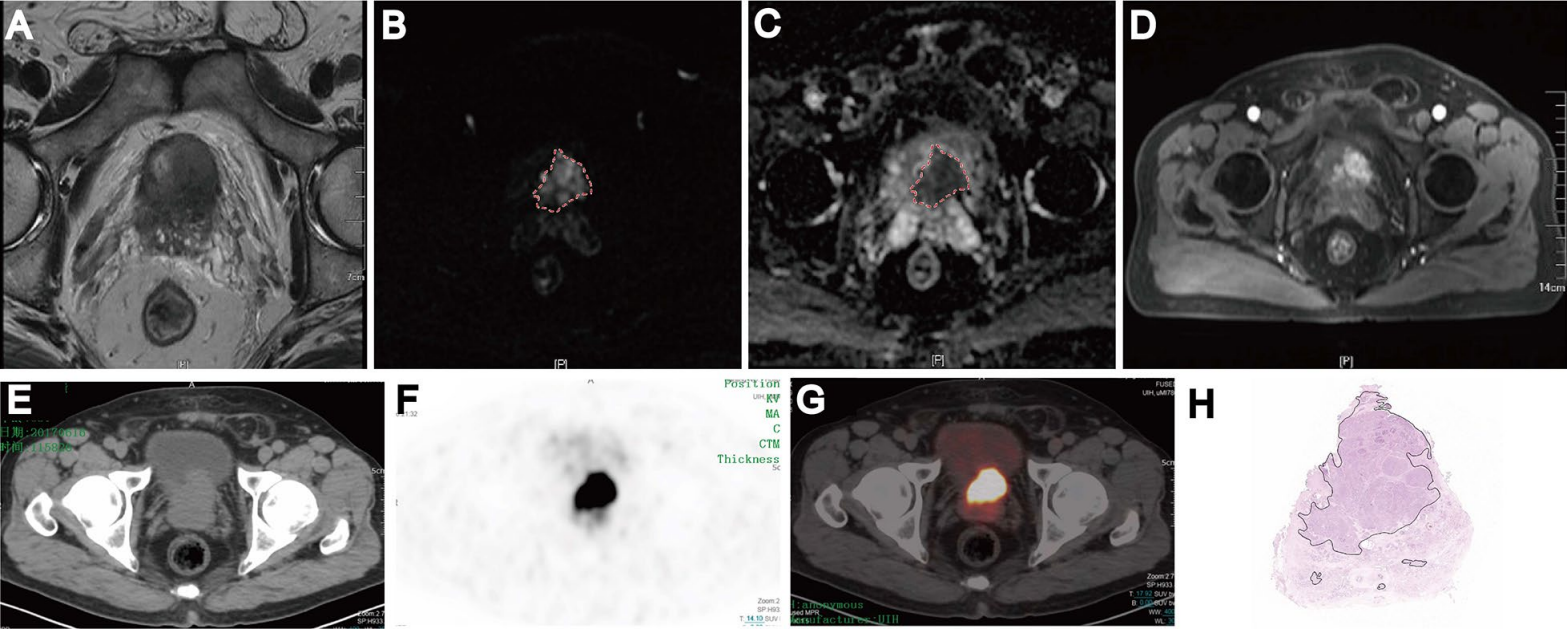
Excellent concordance between mpMRI, ^68^Ga-PSMA-11 PET-CT and whole-gland pathology analysis of a prostate cancer (Gleason score 4 + 5=9/10). T2WI (**A**), DWI (**B**), ADC (**C**), DCE (**D**), CT image (**E**), ^68^Ga-PSMA-11 PET (**F**), ^68^Ga-PSMA-11 PET/CT (**G**) and Histological analyses of whole-gland sections revealed pT3a PCa with Gleason score 9 (4 + 5) (**H**)

Patient characteristics are summarized in Table 1. The mean age of patients was 68.86 years (range 55–82). The mean PSA value was 52.31 ng/mL (range 7.20–151.00). The PSA value of patients with (n = 15) and without (n = 27) LN metastases were 79.63 ng/mL (range 10.95–348.00) and 37.14 ng/mL (range 7.20–128.40), respectively *(P* = 0.029). The Gleason score (*P* = 0.028) and pT stage (*P* = 0.004) at RP in patients with LN metastases were both significantly higher than those without LN metastases. Intraprostatic PCa volume in patients with LN metastases was significantly larger than those without LN metastases (*P* = 0.008). The average maximum SUV of ^68^Ga-PSMA-11 PET-CT positive PCa of patients with and without LN metastases were 13.10 (range 6.12–51.75) and 7.22 (range 5.4–11.2), respectively (*P* < 0.001). Figure 1 displayed the image of preoperative pelvic mpMRI and ^68^Ga-PSMA-11 PET-CT. Figure 1A–D demonstrated the suspicious lesions on different mpMRI sequences (T2WI, DWI, ADC, DCE, respectively). Figure 1E–G presented the ^68^Ga-PSMA-11 PET-CT image, which was in accord with the MRI results. The histological sections of the whole prostate gland confirmed the location of tumor lesions (Fig. 1H). Therefore, ^68^Ga-PSMA-11 PET-CT and pelvic mpMRI succeed on preoperative definite diagnosis and accurate localization of primary PCa in all 42 patients.

**Table 1:** Patient characteristics (n = 42) stratified by nodal status

	Total patients (n = 42)	No LN metastases (n = 27)	LN metastases (n = 15)	*P* value
Age, year, mean, median (range)	68.86, 69 (55–82)	69.26, 69 (55–82)	70.93, 74 (55–79)	0.503
PSA, ng/ml, mean, median (range)	52.31, 37.25 (7.20–348.00)	37.14, 23.50 (7.20–128.40)	79.63, 54.30 (10.95–348.00)	0.029
Gleason score at RP (%)				0.028
3 + 4	9 (21.43)	9 (33.33)	0 (0)	
4 + 3	9 (21.43)	6 (22.22)	3 (20.00)	
≥ 4 + 4	24 (57.14)	12 (44.44)	12 (80.00)	
pT stage at RP, no. (%)				0.004
pT2	11 (26.19)	11 (40.74)	0 (0)	
pT3a	8 (19.05)	6 (22.22)	2 (13.33)	
pT3b	23 (54.76)	10 (37.04)	13 (86.67)	
Intraprostatic PCa size, cm, mean, median (range)	2.72, 2.55 (0.70–5.30)	2.43, 2.30 (0.70–4.30)	3.24, 3.2 (1.00–5.30)	0.828
Intraprostatic PCa volume, ml, mean, median (range)	10.04, 5.46 (0.13–49.00)	6.84, 4.33 (0.126–31.046)	15.86, 9.46 (0.32–48.99)	0.008
LNs removed, no. (%)	621 (100)	381 (61.35)	240 (38.65)	–
LNMs removed, no. (%)	51 (100)	–	51 (100)	–
Short-axis diameter < 5 mm	0	–	0	–
Short axis diameter 5–10 mm	9	–	9	–
Short-axis diameter > 10 mm	42	–	42	–
Intranodal LNM size, mm^a^, mean, median (range)	14.67, 13.00 (7–31)	–	14.67, 13.00 (7–31)	–
Overall LNM size, mm^a^, mean, median (range)	28.87, 30.00 (16–45)	–	28.87, 30.00 (16–45)	–
SUV, maximal LN, mean, median (range)	7.35, 5.40 (4.5–27.72)	–	7.35, 5.40 (4.5–27.72)	–
SUV, maximum PCa, mean, median (range)	9.32, 7.60 (5.4–51.75)	7.22, 6.7 (5.4–11.2)	13.10, 8.12 (6.12–51.75)	< 0.001

*LN* lymph node, *LNM* lymph node metastasis, *PCa* prostate cancer, *PSA* prostate specific antigen, *PSMA* prostate-specific membrane antigen, *RP* radical prostatectomy, *SUV* standardized uptake value

^a^Largest/index lymph node per patient is presented

In total, 621 LNs in all 42 patients were resected with a mean number of 7.095 nodes per patient (range 2–15). Fifteen patients (35.71%) had a pN1 stage, which corresponded to a total of 51 of 621 LNs (8.21%). The numbers of LNMs with short axis diameter 5–10 mm and LNMs with short axis diameter > 10 mm were nine and forty-two, respectively. Fifteen (29.41%), 20 (39.22%), 11 (21.57%), and 5 (9.80%) positive LNs were found along the obturator fossa, external iliac vessels, internal iliac vessels, and common iliac vessels, respectively. No positive LNs were found along the presacral fossa in this study (Table 2).

**Table 2:** Findings in 42 patients at lymphadenectomy

Number of LN	Mean LN/patient	Number of involved LN	OF	EI	II	CI	PSF	Patients with pN1 stage
621	7.095 (2–15)	51 (8.21)	15 (29.41)	20 (39.22)	11 (21.57)	5 (9.80)	0 (0)	15 (35.71)

*LN* lymph node, *pN1* regional LN invaded by micro metastases or ITC, *OF* obturator fossa, *EI* external iliac LN, *II* internal iliac LN, *CI* common iliac LN; *PSF* presacral fossa

**Fig. 2:**
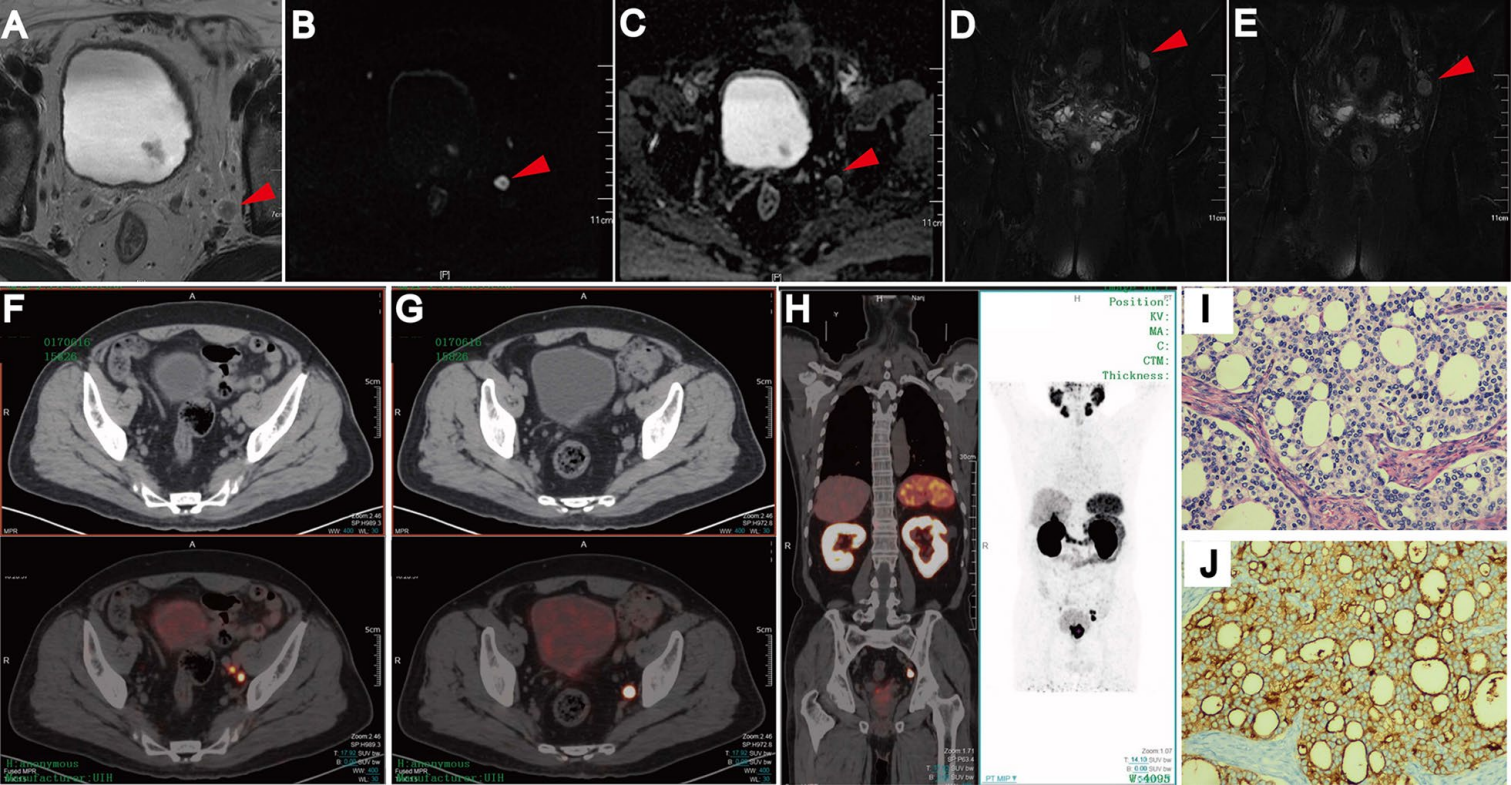
Lymph node metastases on pelvic mpMRI and ^68^Ga-PSMA-11 PET/CT. Axial T2WI (**A**), DWI (**B**), ADC (**C**) and coronal Fat suppression T2WI (**D**, **E**). Fused ^68^Ga-PSMA-11 PET/CT (**F**–**H**) images were taken from left internal iliac and obturator fossa regions with hisopathologically proven disease (HE staining, **I**; PSMA IHC staining, **J**)

Figure 2 displayed positive lymph node at the left internal iliac and obturator fossa on pelvic mpMRI image (A–E, Axial T2WI, DWI, ADC and coronal fat suppression T2WI sequences, respectively) and ^68^Ga-PSMA-11 PET-CT image (F–H). Figure 2I, J presented the histological result of the lymph node, which proved that the lymph node was positive. Table 3 showed the number of correctly and falsely recognized cases, the results of the primary objectives on a patient-based and a LN region-based analysis for ^68^Ga-PSMA-11 PET-CT and mpMRI, respectively (Fig. 2). ^68^Ga-PSMA-11 PET-CT presented a patient-based sensitivity, specificity, PPV and NPV of 93.33, 96.30, 93.33 and 96.30%, respectively. ^68^Ga-PSMA-11 PET-CT showed a LN region-based sensitivity, specificity, PPV and NPV of 96.08, 99.65, 96.08 and 99.65%, respectively. Results of ROC analysis showed that there is significant difference between those two groups (Histopathology positive LN and Histopathology negative LN) (P < 0.001). Area under the curve is 0.993 (95% CI 0.988–0.999) and the best cut-off (SUV max > 3.25) has a sensitivity of 85.7% and a specificity of 99.8%. Moreover, mpMRI demonstrated a patient-based sensitivity, specificity, PPV and NPV of 93.33, 96.30, 87.5 and 96.15%, respectively. Pelvic mpMRI showed a LN region-based sensitivity, specificity, PPV and NPV of 96.08, 99.47, 94.23 and 99.65%, respectively. Table 4 demonstrates the detection results of ^68^Ga-PSMA-11 PET-CT and mpMRI for LNMs by the size of the node based on histopathology. The numbers of the short-axis diameter ≤ 10 mm lymphnodes were detected by ^68^Ga-PSMA-11 PET-CT, mpMRI and pathology were 8, 5 and 9, respectively. The numbers of the short-axis diameter > 10 mm lymphnodes were detected by ^68^Ga-PSMA-11 PET-CT, mpMRI and pathology were 41, 44 and 42, respectively. There was no statistical difference for the detection rate of LNMs according to the diameter of the LNMs between ^68^Ga-PSMA-11 PET-CT (short-axis diameter ≤ 10 mm: 92.75%; short-axis diameter > 10 mm: 97.62%) and mpMRI (short-axis diameter ≤ 10 mm: 92.11%; short-axis diameter > 10 mm: 95.65%) in this study.

**Table 3:** Diagnostic performances of ^68^Ga-PSMA PET/CT and mpMRI, for LN Invasion: per-patient analysis and Per LN-analysis

	PSMA-PET CT		mpMRI	
	Patient analysis	LN analysis	Patient analysis	LN analysis
No. of true-positive cases	14	49	14	49
No. of true-negative cases	26	568	25	567
No. of false-positive cases	1	2	2	3
No. of false-negative cases	1	2	1	2
Sensitivity	(14/15) 93.33%	(49/51) 96.08%	(14/15) 93.33%	(49/51) 96.08%
Specificity	(26/27) 96.30%	(568/570) 99.65%	(26/27) 96.30%	(567/570) 99.47%
PPV	(14/15) 93.33%	(49/51) 96.08%	(14/16) 87.5%	(49/52) 94.23%
NPV	(26/27) 96.30%	(568/570) 99.65%	(15/16) 96.15%	(567/569) 99.65%

*LN* lymph node, *NPV* negative predictive value, *PPV* positive predictive value

**Table 4:** Results of ^68^Ga-PSMA PET/CT and mpMRI in the detection LNMs by the size of the node

	Histopathology positive (n = 51)	Histopathology negative (n = 570)
^68^Ga-PSMA PET/CT positive (n = 51)	49	2
Short-axis diameter < 5 mm	0	0
Short axis diameter 5–10 mm	8	2
Short-axis diameter > 10 mm	41	0
^68^Ga-PSMA PET/CT negative (n = 570)	2	568
Short-axis diameter < 5 mm	0	549
Short axis diameter 5–10 mm	1	19
Short-axis diameter > 10 mm	1	0
mpMRI positive (n = 52)	49	3
Short-axis diameter < 5 mm	0	0
Short axis diameter 5–10 mm	5	1
Short-axis diameter > 10 mm	44	2
mpMRI negative (n = 569)	2	567
Short-axis diameter < 5 mm	0	541
Short axis diameter 5–10 mm	2	26
Short-axis diameter > 10 mm	0	0

*LN* lymph node, *LNM* lymph node metastasis, *PSMA* prostate-specific membrane antigen, *mpMRI* multiparametric magnetic resonance image

## Discussion

LN staging is a crucial element to determine optimal management [14]. Therefore, preoperative accurate assessment of lymph node status is very important in patients with intermediate to high-risk PCa. CT or conventional MRI imaging are commonly used for preoperative LN status evaluation. However, the LN status is largely underestimated with these traditional technique [15, 16]. To date, PLND is considered the most accurate for assessment of nodal involvement, as it provides fresh tissue for pathologic nodal analysis [15]. Nevertheless, this technique is invasive. It is associated with increased lymphocele/lymphedema rates and venous thromboembolism rates [17]. More advanced imaging techniques demonstrated limited sensitivity and specificity such as ^11^C-choline or ^18^F-fluorocholine-based combined PET-CT [16, 18]. Therefore, novel accurate and no invasive imaging techniques might be very important in selecting patients that were suitable for PLND with intermediate to high-risk PCa.

The ideal imaging modality should fulfil some prerequisites: accuracy, availability, reproducibility, cost effectiveness, and efficiency. Recent researches have focused on development of new prostate-specific ligands binding to the PSMA for radionuclide imaging and therapy. The exact role of PSMA in the prostate gland is still discussed; however, it has been shown to be several times more active in PCa cells than in normal prostate cells. Moreover, PSMA expression in PCa is highly increased and has been shown to have prognostic relevance [19]. Recently, PET imaging using PSMA ligands specifically targets PCa cells independent of their metabolic state. ^68^Ga-PSMA-11 PET/CT is a promising technique for primary prostatic lesions, metastasis disease and biochemical recurrence after definitive treatment of acinar prostate cancer [20].

Although the main interest was evaluation of LN involvement, we firstly evaluated patient characteristics stratified by nodal status. In our study, we found that the PSA value, pT stage at RP, intraprostatic PCa volume and SUV, maximum PCa in patients with LN metastases was significantly higher than patients with no LN metastases (Table 1). The results cueing us that patients of PCa with LN metastases had a greater likelihood of malignant behavior.

Budäus et al. [21] retrospectively evaluated the diagnostic performance of preoperative PSMA PET-CT in a cohort of 30 high-risk PCa patients undergoing RP and ePLND. They overall detected 608 LNs containing 53 LNMs. The overall sensitivity, specificity, PPV, and NPV of ^68^Ga-PSMA-11 PET/CT for LNM detection were 33.3, 100, 100, and 69.2%, respectively [21]. However, 42 patients with 621 LNs were resected with a mean number of 7.095 nodes per patient (range 2–15 nodes) in our study. Fifteen patients (35.71%) had a pN1 stage, which corresponds to a total of 51 of 621 LNs (8.21%). Moreover, our study demonstrated a high specificity, sensitivity, PPV, and NPV of ^68^Ga-PSMA-11 PET-CT for the detection of LNMs in men with intermediate and high-risk PCa based on per-patient analysis and per LN-analysis. However, the sensitivity and NPV of ^68^Ga-PSMA-11 PET/CT for LNM detection in our study is significant better than that of the results of Budäus L et al. However, the specificity and PPV of ^68^Ga-PSMA-11 PET/CT for LNM detection in our study is slightly below that of the results of Budäus L et al. This may due to the limited number of positive lymph nodes, especially the number of micro-metastases or isolated tumor cells (ITC) in our study. Therefore, the recently developed functional imaging techniques of ^68^Ga-PSMA-11 PET-CT might add sufficient sensitivity and specificity to overcome the clinically significant N staging problem. Moreover, PSMA-radioguided surgery using a probe intraoperatively is a promising technique that may be able to detect the location of the LN metastases particularly of small lesions more precisely.

MpMRI, including T1- and T2-weighted imaging, DWI, ADC, DCE and other functional sequences. DWI providing information on tissue cellular density and membrane integrity, and DCE MRI, enabling visualization of vascular permeability and perfusion [7]. Currently, mpMRI is the best imaging method for the detection of significant PCa compared to other imaging methods [12, 15]. Moreover, it has shown a high sensitivity and specificity for the detection and characterisation of LN metastases of some types of cancer [22]. Fortunately, the present retrospective evaluation showed promising results for mpMRI in LN staging of intermediate and high-risk PCa with a calculated sensitivity of 93.33% per-patient analysis and 96.08% per-LN analysis, specificity 96.30% per-patient analysis and 99.47% per-LN analysis, PPV 87.5% per-patient analysis and 94.23% per-LN analysis, NPV 96.15% per-patient analysis and 99.65% per-LN analysis, respectively. The specificity, sensitivity, PPV, and NPV of ^68^Ga-PSMA-11 PET-CT is similar to pelvic mpMRI in our study. Thus, there is no incremental value of ^68^Ga-PSMA-11 PET-CT over pelvic mpMRI in terms metastatic lymphnode detection in our study. This excellent result of pelvic mpMRI for lymph node staging in our study may due to high-resolution imaging of 3.0-T MR scanner and multiparametric magnetic resonance sequences (T1- and T2- high-resolution weighted imaging, DWI, ADC, and DCE). Moreover, the sensitivity of sub-centimeter lymph-node in both PSMA PET-CT and mpMRI is high and similar may due to less prevalence of histopathology positive sub-centrimeter lymphnodes in our study. The results in our study indicated that ^68^Ga-PSMA-11 PET-CT and mpMRI both appears to provide helpful additional information in the staging of PCa with intermediate to high metastatic risk. Our study is an important research on the assessment of LN status preoperative. However, further studies are needed to investigate the possible contribution of ^68^Ga-PSMA PET-CT to mpMRI for the primary detection of PCa.

There are limitations associated with this study. First, the selection of patients with intermediate to high probability for LNMs might have overestimated the diagnostic performances of ^68^Ga-PSMA-11 PET-CT and mpMRI. Second, presacral fossa LNs were not removed, which might have influenced the sensitivity. Third, since all PET-CT and mpMRI scans were reported by experienced physicians, the present results may not be directly adaptable to clinical practice. Finally, as a small retrospective single center study the results should be confirmed in larger, prospective and multicenter settings.

## Conclusions

^68^Ga-PSMA-11 PET-CT and mpMRI in our study appears to provide helpful additional information in the staging of PCa with intermediate to high metastatic risk. Both mpMRI and ^68^Ga-PSMA-11 PET-CT have a high specificity, sensitivity, PPV, and NPV for the detection of LNMs in men with intermediate and high-risk PCa. Preoperative ^68^Ga-PSMA-11 PET-CT-guided region-based LN dissection represents a suitable approach since it is less time consuming and easier to perform without losing information. However, despite excellent performance, it cannot replace mpMRI that remains excellent for tumoral localization, local evaluation, and lymph node staging.

## Abbreviations

mpMRI: multiparametric magnetic resonance imaging
PCa: prostate cancer
RP: radical prostatectomy
PLND: pelvic lymph node dissection
PSMA: prostate-specific membrane antigen
^68^Ga-PSMA-11: [^68^Ga] Glu-urea-Lys(Ahx)-HBED-CC
SUV: standardized uptake value
PPV: positive predictive value
NPV: negative predictive value
